# Genetic Diversity of Five Local Swedish Chicken Breeds Detected by Microsatellite Markers

**DOI:** 10.1371/journal.pone.0120580

**Published:** 2015-04-09

**Authors:** Abiye Shenkut Abebe, Sofia Mikko, Anna M. Johansson

**Affiliations:** Department of Animal Breeding and Genetics, Swedish University of Agricultural Sciences, Uppsala, Sweden; Temasek Life Sciences Laboratory, SINGAPORE

## Abstract

This study aimed at investigating the genetic diversity, relationship and population structure of 110 local Swedish chickens derived from five breeds (Gotlandshöna, Hedemorahöna, Öländsk dvärghöna, Skånsk blommehöna, and Bohuslän- Dals svarthöna, in the rest of the paper the shorter name Svarthöna is used) using 24 microsatellite markers. In total, one hundred thirteen alleles were detected in all populations, with a mean of 4.7 alleles per locus. For the five chicken breeds, the observed and expected heterozygosity ranged from 0.225 to 0.408 and from 0.231 to 0.515, with the lowest scores for the Svarthöna and the highest scores for the Skånsk blommehöna breeds, respectively. Similarly, the average within breed molecular kinship varied from 0.496 to 0.745, showing high coancestry, with Skånsk blommehöna having the lowest and Svarthöna the highest coancestry. Furthermore, all breeds showed significant deviations from Hardy-Weinberg expectations. Across the five breeds, the global heterozygosity deficit (F_IT_) was 0.545, population differentiation index (F_ST_) was 0.440, and the global inbreeding of individuals within breed (F_IS_) was 0.187. The phylogenetic relationships of chickens were examined using neighbor-joining trees constructed at the level of breeds and individual samples. The neighbor-joining tree constructed at breed level revealed two main clusters, with Hedemorahöna and Öländsk dvärghöna breeds in one cluster, and Gotlandshöna and Svarthöna breeds in the second cluster leaving the Skånsk blommehöna in the middle. Based on the results of the STRUCTURE analysis, the most likely number of clustering of the five breeds was at K = 4, with Hedemorahöna, Gotlandshöna and Svarthöna breeds forming their own distinct clusters, while Öländsk dvärghöna and Skånsk blommehöna breeds clustered together. Losses in the overall genetic diversity of local Swedish chickens due to breeds extinction varied from -1.46% to -6.723%. The results of the current study can be used as baseline genetic information for genetic conservation program, for instance, to control inbreeding and to implement further genetic studies in local Swedish chickens.

## Introduction

Intensive selection and crossbreeding in selected chicken breeds have been practiced over a long time [1] and has subsequently led to developed commercial strains that dominate the present commercial poultry industry. Introduction of these strains, however, threatened the existence of native chicken breeds [1, 2, 3]. This is because commercialization of chicken breeding favored the use of highly productive chicken breeds, and consequently led to lower population sizes of low performing native breeds [4]. Moreover, most of the genetic and phenotypic studies have focused on elite commercial chicken breeds kept in industrialized countries [5]. Due to poor commercial performances, native chicken breeds in many countries are often ignored and far less attention is given to genetic conservation of these resources compared to other livestock species such as cattle and sheep [1, 6, 7].

Today there are eleven local Swedish chicken breeds present. Most of the local breeds became threatened by extinction when the commercially international breeds became more common. Typically, only one or few populations with a small number of chickens remained when the Swedish association for local poultry (Svenska Lanthönsklubben) rescued them. The association is still working on maintaining the local chickens in the form of live gene bank. Studies on local Swedish chickens are limited. One breed was included in a study of dermal hyperpigmentation [8]. The mtDNA D-loop was recently sequenced in nine of the breeds. There is limited mtDNA diversity with 7 different haplotypes and several breeds having the same haplotype [9]. Due to the limited mtDNA diversity, it is necessary to analyse autosomal markers in the Swedish local breeds, in order to study diversity and the relatedness between breeds. So far, there is no information about the genetic diversity of local Swedish chickens. Therefore, it is of special interest to identify the genetic variability among breeds to strengthen the genetic conservation program implemented by the Swedish association for local poultry.

Currently, microsatellite loci are the method of choice to study the genetic diversities within and between populations because they are highly polymorphic, show co-dominant inheritance, found to be abundant and evenly distributed throughout the genome [2, 4, 10, 11]. So far, many studies have been conducted to assess chickens genetic diversity using microsatellite markers and the reported results are clear evidences of the usefulness of these panels for biodiversity studies [2, 7, 12]. Using microsatellites on our samples from Swedish breeds also allow comparison with published studies of breeds in other countries.

**Fig 1:**
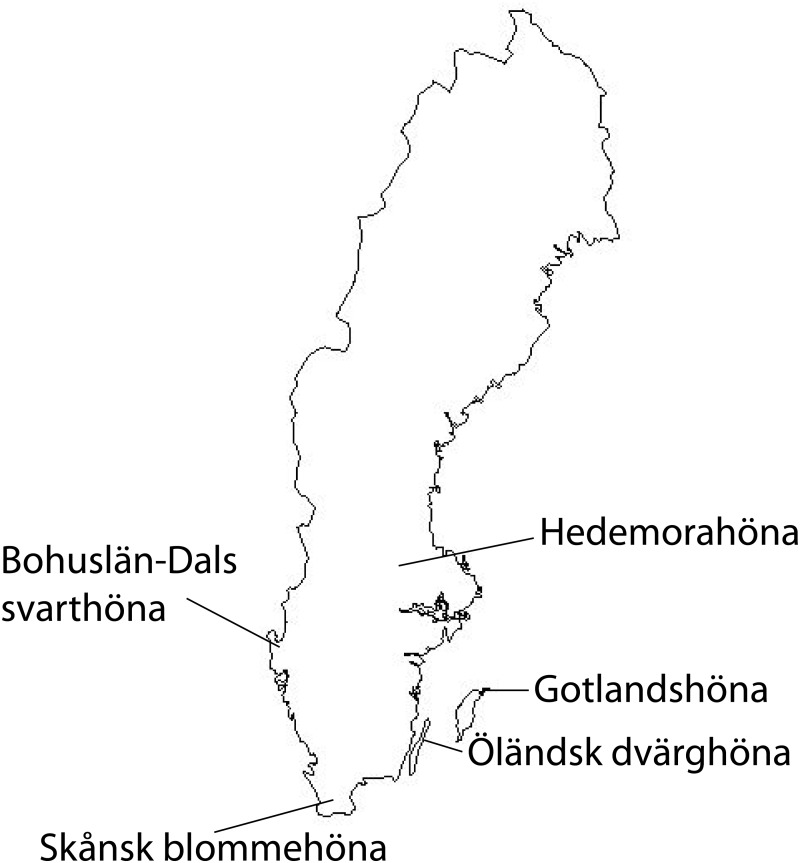
Map of Sweden with the geographic origin of the five breeds indicated.

The present study used data obtained from five of the eleven local Swedish chicken breeds. Since these breeds originate from different parts of Sweden (Fig 1) and have been naturally selected for traits that fit their local environment, they might have possessed unique genetic characteristics. Knowledge on the genetic diversity and breed structure; *i)* provides us with more insight about the differences and similarities between breeds, *ii)* can be used as a basic input for future improvement of breeds and to implement effective breed conservation program. This study aimed at investigating the genetic diversity, genetic relationship and population structure of five local Swedish chicken breeds using 24 microsatellite markers.

## Materials and Methods

### Samples collection and DNA extraction

Blood samples were obtained from 110 local Swedish chickens derived from five breeds: Gotlandshöna (N = 33, three flocks), Hedemorahöna (N = 36, three flocks), Öländsk dvärghöna (N = 17, two flocks), Skånsk blommehöna (N = 10, two flocks) and Bohuslän-Dals svarthöna (mentioned throughout the paper with its common name Svarthöna) (N = 14, two flocks). Chickens were sampled from members of the Swedish association for local poultry. The samples were taken by visits to the owners of the birds or taken by a local veterinarian. Ethical permission was obtained prior to the sampling (see ethics statement below). Gotlandshöna originate from two farms on the island Fårö close to the island Gotland in the Baltic sea. Hedemorahöna originate from one flock in the area close to Hedemora in the county Dalarna. Öländsk dvärghöna originates from two villages on the north part of the island Öland in the Baltic sea. Skånsk blommehöna originate from three villages in the middle part of the county Skåne in southern Sweden. Svarthöna originate from northern Bohuslän in western Sweden (close to the Norwegian border). Genomic DNA for each sample was extracted using QIAamp DNA Blood Midi Kit (QIAGEN, Hilden, Germany). DNA from some samples were prepared manually and from some it was prepared with the QIASymphony robot.

### Ethics statement

Ethical permission (number C247/6) for the collection of blood samples was obtained from the Uppsala ethical board for animal research (name in Swedish: Uppsala djurförsöksetiska nämnd) prior to the collection of samples.

### Microsatellite Genotyping

Twenty-four microsatellite loci were used to assess the DNA polymorphism. All of the markers are part of the 30 microsatellites recommended by the International Society of Animal Genetics (ISAG)-FAO to study the genetic diversity of chickens [5].

Polymerase chain reaction (PCR) amplifications were performed based on multiplex PCR techniques using the QIAGEN hot star enzyme (QIAGEN, Valencia, CA, USA) and we optimized the multiplexes manually. The 24 microsatellite markers were grouped into six multiplexes each with three to five pairs of primers per reaction plate. A final volume of 10 μl multiplex PCR master-mix contained: 10x PCR buffer with MgCl_2_, dNTPs (25 mM), QIAGEN HotStar Tag (5 U/μl), distilled water, fluorescently labeled forward and unlabeled reverse primers each with 10 μM concentration and genomic DNA (25 ng/μl). Amplification using the thermo-cycler ABI9700 was carried out with an initial incubation and enzyme activation of 95° for 5 min., followed by 35 cycles of denaturation at 90° for 30 sec., primer annealing at 55° for 45 sec. and extension of 72° for 30 sec. and lastly a final extension of 72° for 15 min. After amplification, fragment analysis was conducted using 3500xL genetic analyzer (Applied BioSystems). The GeneMapper 5 computer Software package (Applied BioSystems) was used to determine the fragment sizes and allele calling by comparing with known internal size standard.

### Genetic diversity estimates

Basic measures of genetic diversity, such as total number of alleles, allele frequencies, mean number of alleles, observed and expected heterozygosity [13] were computed using FSTAT version 2.9.3 [14] and Excel Microsatellite Toolkit. Polymorphism information content (PIC), a measure of how microsatellite loci are informative in relation to expected heterozygosity [15], was calculated for each marker using Cervus version 3.0 software [16]. Hardy-Weinberg exact tests per breed and locus were performed using the Markov chain algorithms with a dememorization step of 10,000 for 100 batches with 5,000 iterations per batch as implemented in Genepop version 4.2 [17]. Wright’s Fstatistics (F_IT_, F_ST_ and F_IS_) were computed according to Weir and Cockerham [18] using FSTAT software.

### Genetic relationships

The genetic relationship of local Swedish chicken breeds was studied using two methods. First, we computed Nei’s [19] standard genetic distance from allele frequencies, and then bootstrapped 1000 times across all loci using PHYLIP version 3.9 software [20]. Subsequently, the neighbor joining method implemented in PHYLIP was used to construct the consensus phylogenetic tree at the level of individuals and breeds. In the second method, genetic structure of the studied chicken breeds was inferred from multi-locus genotype data using a Bayesian based approach employed in STRUCTURE version 2.3.4 software package [21]. The analysis was carried out using an admixture model with independent allele frequencies between breeds [21, 22]. We ran the STRUCTURE analysis with an initial length of 20,000 burn-in periods followed by 100,000 MCMC (Marco Chain Monte Carlo) repeats for K (number of clusters) ranging from 2 to 8. For each value of K, 100 independent runs were performed. Pairwise comparisons of the 100 solutions were carried out in a greedy algorithm implemented in CLUMPP version 1.1.2 software [23]. Finally, clusters with the highest average pairwise similarity index (H) were converted into postscript file using DISTRUCT version1.1 software [24] and the different clusters were visualized using Ghost view.

The most likely number of clusters (*Δ*K) was calculated following the equation proposed by [25].

$$\;\Delta \text{K}=\text{m}\left(\left|L^{\prime \prime }\left(\text{K}\right)\right|\right)/\text{s}\left[\text{L}\left(\text{K}\right)\right]$$

where *m*(|*L"*(*K*)|) was the mean absolute value of the second order rate of change of the estimated log likelihood of the data, while *s*[*L*(*K*)] was the standard deviation of the estimated log likelihood.

### Genetic contributions of breeds

The genetic contribution of breeds to the total genetic diversity was quantified following the method proposed by Caballero and Toro [26]. The method involves partitioning of the total genetic diversity into the within and between breed diversity. This was done by removing one or more breeds at a time from the population and then, quantifying the within and between breed contributions based on the molecular co-ancestry information using Molkin version 3.0 software [27]. Positive contributions to diversity from any breed using Caballero and Toro [26] method mean that the overall diversity increased because of the remaining breeds, as a result, the assessed breed would be less preferred, for instance, for genetic conservations. To avoid biases due to variations in sample sizes between breeds, we generated 100 samples with 50 individuals per population using the bootstrapping methods implemented in Molkin version 3.0.

## Results and Discussion

### Genetic diversity within and among breeds

One hundred thirteen alleles were identified in the five local Swedish chicken breeds assessed at 24 microsatellite loci. The number of alleles per locus ranged from two at MCW0098 to eight at MCW0183, with an average of 4.7 (Table 1).

**Table 1:** Number of alleles (NA), polymorphism information content (PIC), observed (HO) and expected (HE) heterozygosity with standard deviation of the mean values within parenthesis and Hardy Weinberg (HW) tests.

Loci	NA	PIC	HO	HE	HW
LEI0094	7	0.684	0.291	0.723	*
ADL0268	5	0.597	0.344	0.623	*
MCW0248	5	0.326	0.203	0.324	**
MCW0216	4	0.480	0.301	0.541	***
ADL0278	7	0.643	0.439	0.686	NS
MCW0295	6	0.687	0.300	0.743	***
MCW0081	6	0.579	0.445	0.671	*
MCW0069	6	0.739	0.359	0.767	***
MCW0034	6	0.754	0.481	0.798	**
MCW0222	4	0.223	0.301	0.297	NS
MCW0111	4	0.481	0.299	0.628	NS
MCW0037	5	0.576	0.747	0.635	NS
LEI0166	4	0.619	0.451	0.676	NS
ADL0112	4	0.594	0.297	0.643	NS
MCW0014	4	0.463	0.048	0.522	***
MCW0183	8	0.536	0.190	0.513	***
MCW0123	6	0.679	0.299	0.741	NS
MCW0165	3	0.569	0.080	0.657	**
MCW0020	4	0.669	0.395	0.734	NS
MCW0104	3	0.544	0.201	0.634	NS
MCW0078	3	0.308	0.295	0.335	NS
MCW0067	4	0.545	0.259	0.651	***
MCW0330	3	0.566	0.289	0.667	***
MCW0098	2	0.374	0.264	0.477	NS
Mean (Std. dev.)	4.7 (1.49)	0.551 (0.13)	0.316 (0.14)	0.612 (0.139)	

*p<0.05;

**p<0.01;

***p<0.001;

*NS = non-significant*

The mean polymorphism information content of loci was 0.551, ranging from 0.223 to 0.754 (Table 1). According to Botstein *et al*. [28], all microsatellite loci included in the present study were reasonably informative except MCW0222 locus. However, within breeds, many loci were monomorphic. Nine loci (LEI0094, ADL0268, MCW0248, MCW0222, ADL0112, MCW0014, MCW0165, MCW0078 and MCW0098) for Svarthöna, four loci (MCW0111, MCW0014, MCW0183 and MCW0123) for Öländsk dvärghöna, ADL0278 for Hedemorahöna and MCW0098 locus for Skånsk blommehöna were found monomorphic (data not shown). The monomorphic loci in a breed could be due to limited sample size, high inbreeding within population, presence of null alleles or lack of effectiveness of microsatellite loci in a wide spectrum of chicken populations. All the microsatellite loci used in the present study are members of the 30 loci recommended by ISAG-FAO for genetic diversity studies in chickens.

The observed proportions of heterozygosity for the 24 loci ranged from 0.048 at MCW0014 to 0.747 at MCW0037, with the average being 0.316. In turn, expected heterozygosity varied from 0.297 at MCW0222 to 0.798 at MCW0034, and the average across all loci was found to be 0.612 (Table 1). Values of observed and expected heterozygosity varied among loci. Because the evolutionary forces, such as mutation and random genetic drift, may affect loci differently so that it eventually changes the amount of heterozygosity [29].

Genetic diversity measures within the five chicken breeds are summarized in Table 2. The mean number of alleles per locus ranged from 1.9 for Svarthöna to 3.2 for Skånsk blommehöna breed. Svarthöna breed also showed the lowest observed and expected heterozygosity, 0.225 and 0.231 respectively. Such low heterozygosity is likely the result of mating between genetically related individuals, as the highest within breed molecular coancestry was observed in Svarthöna breed. The average molecular kinship between parents is equal to the level of inbreeding in the offspring [30]. It should be noted that the high average molecular kinship observed in Svarthöna is expected to result in a high level of inbreeding in the next generation of this breed. Skånsk blommehöna, however, showed better within breed genetic diversity, with observed (0.408) and expected (0.515) heterozygosity. This is in agreement with the mtDNA results where Skånsk blommehöna showed the highest diversity with 4 haplotypes present among the five individuals that were seqiuenced, whereas most other breeds had only a single haplotype [9]. Although population size is quite small for all local Swedish chicken breeds, the Skånsk blommehöna breed has broader genealogical origin. Skånsk blommehöna originates from flocks in three different villages, whereas the now living birds of the other breeds originate from only one or two villages. This could be a reason for its higher genetic diversity compared to the remaining breeds.

**Table 2:** Mean number of alleles (MNA) per locus, observed (HO) and expected (HE) heterozygosity, Hardy-Weinberg test (HW) and mean molecular kinship within breed.

Breeds	MNA ± Std. dev.	HO ±Std. dev.	HE ±Std. dev.	HW	Mean molecular kinship
Gotlandshöna	3.0 ± 0.9	0.318 ± 0.017	0.383 ± 0.042	***	0.600
Hedemorahöna	2.8 ± 0.9	0.306 ±0.016	0.400 ± 0.038	***	0.599
Öländsk dvärghöna	2.2 ± 0.9	0.322 ± 0.023	0.380 ± 0.040	**	0.615
Skånsk blommehöna	3.2 ± 1.1	0.408 ± 0.032	0.515 ± 0.038	***	0.496
Svarthöna	1.9 ± 0.8	0.225 ± 0.023	0.231 ± 0.045	**	0.754

**p<0.01;

***p<0.001

Generally, the within breed genetic diversity of the studied chicken breeds can be regarded as low. This is evidenced by low heterozygosity estimates, small number of alleles detected per locus and high within breed molecular coancestry (Table 2). Furthermore, all of the five breeds were significantly deviated from Hardy-Weinberg expectations (Table 2) and across all breeds, more than half of the loci showed significant deviation from Hardy-Weinberg (Table 1). Indeed, local Swedish chickens have not been selected for specific traits, thus low within breed diversity may be associated with small effective population size of the breeds and lack of effective breeding strategies, for instance, to minimize within breed coancestry. Keeping small isolated flocks over many generations may result in loss of heterozygosity due to the high chances of random genetic drift and inbreeding [31, 32]. In addition, heterozygosity deficit could also occur due to null allele effects, a situation in which the genotyping assay failed to detect alleles due to mutations in the primer binding sites [33]. In the present study, however, no locus was fixed in all breeds. The amount of heterozygosity estimated for local Swedish chickens is fairly similar to the corresponding estimates for six local Italian chicken breeds [34]. Other studies (e.g. for local Zimbabwe chickens [35]; local Turkish chickens [12]; local Vietnamese chickens [36]) reported relatively larger heterozygosity estimates for different local chicken breeds using microsatellite loci.

The fixation indices (F_IT_, F_ST_ and F_IS_) per locus across the five breeds are depicted in Table 3. With a mean value of 0.545, the global heterozygosity deficit of individuals within the total population (F_IT_) was significantly high (P<0.001). Fixation index of subpopulation in relation to the total population (F_ST_) per locus varied from 0.079 at MCW0248 to 0.891 at MCW0104 locus, with the mean being 0.440 (P<0.001). This indicates that about 44% of the total genetic variation in local Swedish chicken population is explained by between breed differences. Nineteen of the 24 loci contributed significantly to the genetic differentiation among breeds. The mean F_ST_ in the present study was approximately equivalent to mean F_ST_ values of 0.437 and 0.429 reported by Zanetti *et al*. [34] for six local Italian chicken breeds and Tadano *et al*. [37] for seven Japanese native chicken breeds, respectively. The average inbreeding coefficient of individuals within the subpopulations, measured as F_IS_ value, across the 24 loci was 0.187 (P<0.001). This shows about one-third of the global heterozygosity reduction was due to the within breed deficit. Nine markers revealed significant deficit of heterozygosity, but only three loci (MCW0037, MCW0078 and MCW0222) showed excess of heterozygosity (Table 3).

**Table 3:** Fixation indices (F_IT_, F_ST_ and F_IS_) calculated according to Weir and Cockerham [17] across five local Swedish chicken breeds.

Loci	F_IT_ *(F)*	F_ST_ *(θ)*	F_IS_ *(f)*
LEI0094	0.519	0.429*	0.083
ADL0268	0.687**	0.547**	0.242
MCW0248	0.283	0.079**	0.222
MCW0216	0.675***	0.485***	0.329*
ADL0278	0.557***	0.523**	0.077
MCW0295	0.500***	0.293*	0.281**
MCW0081	0.388***	0.235	0.226
MCW0069	0.612***	0.413***	0.348**
MCW0034	0.444***	0.403***	0.072
MCW0222	0.043	0.120	-0.083
MCW0111	0.581**	0.499*	0.168***
MCW0037	-0.125*	0.204**	-0.400
LEI0166	0.650*	0.621	0.095
ADL0112	0.656***	0.609***	0.111***
MCW0014	0.898***	0.318	0.848***
MCW0183	0.715***	0.669**	0.287*
MCW0123	0.592***	0.511**	0.172
MCW0165	0.870***	0.731***	0.563*
MCW0020	0.512***	0.407**	0.172***
MCW0104	0.909***	0.891***	0.125
MCW0078	0.105	0.337***	-0.323
MCW0067	0.773**	0.643***	0.335
MCW0330	0.673***	0.419**	0.487
MCW0098	0.282	0.272	0.038
Mean	0.545 ***	0.440***	0.187***

* p<0.05;

** p<0.01;

*** p<0.001.

### Phylogenetic relationships

**Fig 2:**
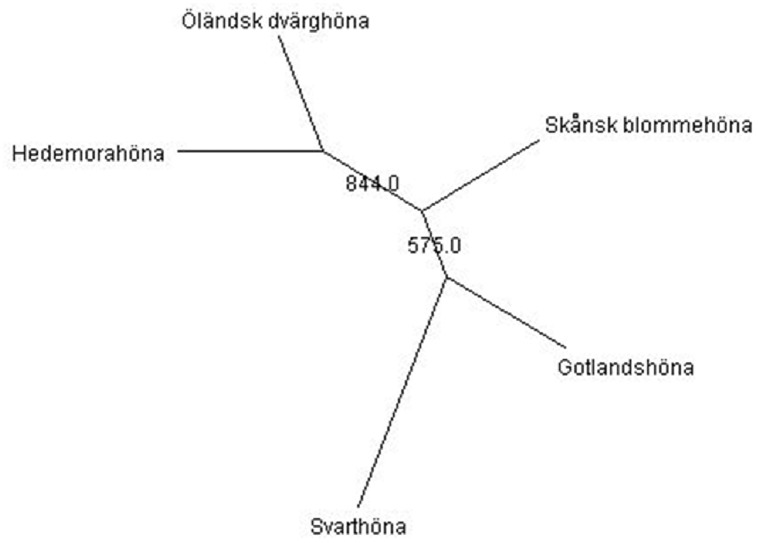
Unrooted neighbor-joining consensus tree constructed using Nei’s genetic distance of five local Swedish chicken breeds. The numbers on the branches show the frequency of occurrences of the associated branch from 1000 bootstrapping.

**Fig 3:**
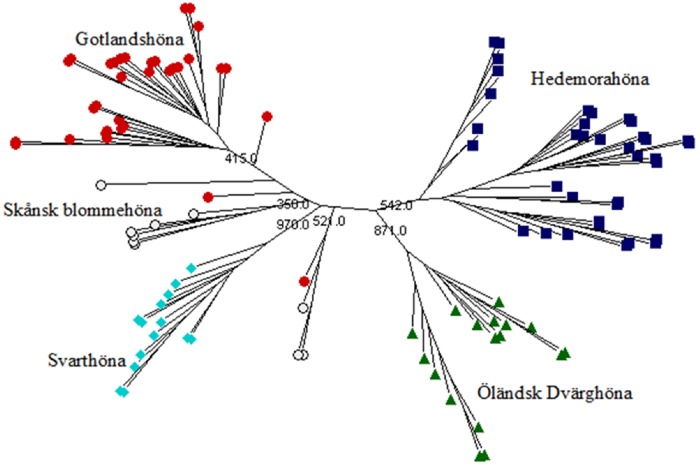
Neighbor-joining tree of individual chickens. Bootstrap values are shown partially. Each breed is marked with a specific color.

The consensus neighbor-joining tree derived from Nei’s [19] standard genetic distance of five local Swedish chicken breeds is given in Fig 2. The phylogenetic tree revealed two main clusters, with Hedemorahöna and Öländsk dvärghöna breeds in one cluster, and Gotlandshöna and Svarthöna breeds in the second cluster. Such clustering of breeds into two groups highlighted the presence of clear genetic separation between breeds at different groups. This is also in agreement with the high mean F_ST_ estimated across the 24 loci, showing significant genetic differentiations between breeds. A neighbor-joining tree constructed using individual samples (Fig 3) showed that all individuals from Hedemorahöna, Öländsk dvärghöna and Svarthöna breeds clustered to their breed of origin. Similarly, all but two individuals for the Gotlandshöna were clustered to the predefined breed. Although not supported by high bootstrap values, individuals of the Skånsk blommehöna breed did not follow uniform clustering as shown in Fig 3. Based on neighbor-joining tree clustering assessment, it may be difficult to identify admixture or migrant individuals that are not clustered to their breed category. This is because the neighbor-joining tree uses distance matrices of individuals compressed at breed level (Fig 2) and in addition it does not show the fraction of genome shared in individuals that have recent ancestors in more than one breed [38].

### Breeds structure and individual’s assignment

**Fig 4:**
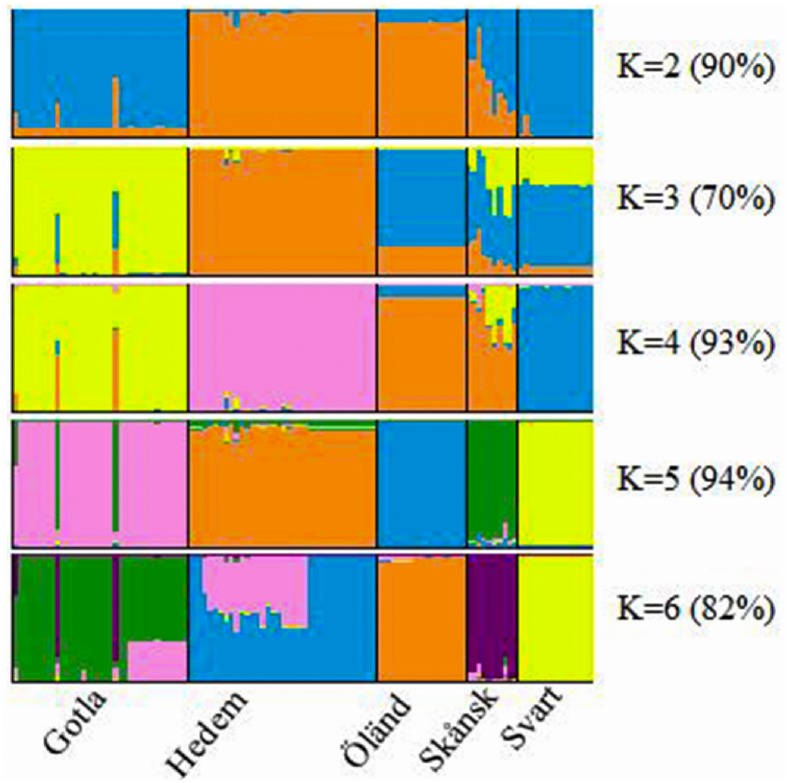
STRUCTURE Clustering of five local Swedish chicken breeds. Gotla = Gotlandshöna; Hedem = Hedemorahöna; Öland = Öländsk dvärghöna; Skånsk = Skånsk blommehöna; Svart = Svarthöna. Numbers in the parenthesis indicate the average similarity index between individuals assigned into the same cluster

The genetic structure of breeds was studied using a model-based clustering approach that assigns individuals into one or more populations probabilistically based on the allele frequencies detected at different loci. The results of STRUCTURE clustering from K = 2 to 6 are displayed in Fig 4. When low value of K (i.e. K = 2) was assumed, individuals were clustered to the putative breeds in a similar way to the results shown in the neighbor-joining tree in Fig 1. At K = 3, Gotlandshöna and Hedemorahöna breeds clustered independently, and thus can be regarded as genetically distinct breeds. Subsequently, at K = 4, only Öländsk dvärghöna and Skånsk blommehöna breeds failed to show separate clusters, but at K = 5, all the five breeds were placed into separate clusters. Mean log-likelihood of the data steadily increased from K = 2 to K = 4 and displayed plateau appearance without significant changes from K = 5 to K = 8 (data not shown). Based on the method proposed by Evanno *et al*. [25], the most likely number of clusters (Δ K) that captures most of the genetic structure of the studied breeds was at K = 4. Kalinowski [39] showed that STRUCTURE based clustering of individuals could potentially be affected by sample size. Most probably, the effect of small sample size may be the reason why the Skånsk blommehöna breed did not show distinct cluster until K is equal to the number of breeds. However, according to Rosenberg et al. [38], the sample size of Skånsk blommehöna breed and the number of loci used are sufficient to show at least 90% clustering success rate. Notably, three individuals within the Gotlandshöna breed assigned in more than one cluster, showing they were admixed. The proportion of membership coefficients shared by different clusters evidenced this.

### Genetic contribution of breeds

The genetic contribution of five local Swedish chicken breeds estimated using Caballero and Toro [26] approach is given in Table 4. Extinction of one breed from the total population resulted in loss of the overall genetic diversity, which ranged from -1.46% to -6.723% when the Skånsk blommehöna and Gotlandshöna were removed, respectively. The removal of Svarthöna breed increased internal diversity of the studied local chicken population. This could be attributed to the low within breed diversity and high molecular coancestry of individuals in Svarthöna breed (Table 2). Except the Skånsk blommehöna breed, removal of any of the remaining breeds resulted in loss of between breed diversity of the total population. This can be explained by relatively large mean genetic distance estimated between breeds. Despite the high within breed genetic diversity of Skånsk blommehöna breed (Table 2), its contribution to the aggregate genetic diversity was the lowest. In this regard, it is particularly important to give due consideration for the within breed variability to avoid conserving inbred populations and prevent accumulation of extreme alleles [26, 34].

**Table 4:** Loss or gain (%) of the total genetic diversity of local Swedish chickens when one of the breed was removed from the population based on the method developed by Caballero and Toro [25].

Breeds	Genetic diversity(%)	within breed(%)	Between breed(%)	Loss (-) / gain (+)(%)
Gotlandshöna	0.565	-0.910	-5.813	-6.723
Hedemorahöna	0.578	-1.338	-3.352	-4.690
Öländsk dvärghöna	0.585	-0.103	-3.445	-3.548
Skånsk blommehöna	0.597	-2.180	0.720	-1.460
Svarthöna	0.595	3.581	-5.356	-1.775

### Advice for conservation

We have shown that these microsatellites work well to study diversity in the Swedish breeds. Based on our findings, owners and The Swedish association for local poultry can prioritize which breeds that are most important to focus the conservation efforts based on the within, between or overall contributions to the gene pool. Since this study have shown that there is low number of alleles within all the five studied breeds, breeding programs for these breeds should focus on preventing loss of further alleles. It is also important to increase the observed heterozygosity by reducing inbreeding. Since there are no pedigree record for individuals (although there is records on flock level on the exchange of birds between flocks), molecular markers can be used instead of pedigrees to access kinship between individuals. Ideally, if a large proportion of the birds in a breed are genotyped, the decision of purchasing of a new breeding animal to a flock can be based on the molecular kinship, so that an animal that have low molecular coancestry to the existing animals in the flock is chosen. Furthermore, sustainable breed conservation program requires huge resources to be invested over long period. Therefore, on top of microsatellite loci based information, breed evaluation for conservation should address other issues such as adaptability of breeds, economic importance and social values of the breeds [26, 40].

## Conclusion

In conclusion, the five local Swedish chicken breeds showed low within breed genetic diversity but considerable variations exist between breeds. Relatively high molecular coancestry observed within breeds could potentially increase the level of inbreeding in subsequent generations. Thus, our results highlighted the importance of implementing effective breeding strategies, for example designing breeding programs and applying mating based on minimum molecular coancestry, to prevent further losses in genetic diversity.

## Supporting Information

S1 File Contains microsatellite genotypes for the Swedish chicken samples used in this paper. (XLSX)
